# Evaluation of the Effectiveness of Injectable Platelet-Rich Fibrin as an Adjuvant to One-Stage Full-Mouth Disinfection in Patients with Stage II and III Periodontitis: Protocol for a Randomized Clinical Trial

**DOI:** 10.2196/81032

**Published:** 2026-02-19

**Authors:** Mahima Kothekar, Pavan Bajaj, Sneha Dare, Shivani Thakre

**Affiliations:** 1 Department of Periodontics and Implantology, Sharad Pawar Dental College, Datta Meghe Institute of Higher Education and Research, Sawangi (Meghe), Wardha, Maharashtra, 442001, India, 918805598774

**Keywords:** one-stage full-mouth disinfection, OS-FMD, full mouth disinfection, FMD, injectable platelet rich fibrin, i-PRF, adjuvant

## Abstract

**Background:**

Periodontitis is a chronic inflammatory disease that leads to the progressive destruction of the tooth-supporting structures, including the periodontal ligament, alveolar bone, and gingival tissues, leading to tooth mobility, ultimately resulting in potential tooth loss if left untreated. The new classification of periodontitis helps in establishing an appropriate diagnosis and planning treatment according to disease severity. One-stage full-mouth disinfection (OS-FMD), using chlorhexidine, has shown better outcomes than traditional quadrant-wise therapy. Platelet-Rich Fibrin offers enhancement in healing outcomes and regeneration due to sustained release of growth factors. Injectable platelet-rich fibrin (i-PRF) shows promising results in promoting tissue regeneration, reducing inflammation, and improving periodontal therapy outcomes. To date, no clinical study has been carried out for the assessment of the efficacy of i-PRF as an adjuvant in OS-FMD.

**Objective:**

This study aims to assess the efficacy of i-PRF as an adjuvant in OS-FMD therapy based on its clinical outcomes in terms of plaque index (PI), papillary bleeding index (PBI), probing depth (PD), and clinical attachment loss (CAL) in patients with stage II and stage III periodontitis.

**Methods:**

This randomized clinical trial will include 26 systemically healthy patients diagnosed with stage II and III periodontitis, selected from the Outpatient Department of Periodontics at Sharad Pawar Dental College, Sawangi (Meghe), Wardha. Participants will be randomly assigned to one of 2 groups using a parallel-arm design to ensure unbiased allocation. The control group will undergo OS-FMD therapy involving subgingival scaling and root planing for the entire dentition, which will be performed within 24 hours, supplemented by application of chlorhexidine intraorally, including mouth rinsing, pocket irrigation, and tongue cleansing. The test group will receive the same OS-FMD protocol as the control group, in addition to subgingival delivery of i-PRF in all periodontal pockets 1 week post therapy. Assessment of clinical parameters, including PI, PBI, PD, and CAL, will be done at baseline, 3 months, and 6 months. To evaluate intragroup and intergroup differences, statistical analysis will be conducted using appropriate methods, including the Wilcoxon signed-rank test. A *P* value <.05 will be considered statistically significant.

**Results:**

The study was enrolled in June 2025 and is scheduled to conclude post assessments and analyses by the end of 2026. The accessibility of the study results is anticipated in early 2027.

**Conclusions:**

This study underscores the effectiveness of i-PRF as an adjuvant in OS-FMD therapy on the basis of assessment of clinical parameters. We hypothesize that the use of i-PRF as an adjuvant to OS-FMD will result in superior clinical outcomes beyond the antimicrobial benefits achieved with chlorhexidine in standard OS-FMD, as evidenced by CAL gain, reduction in PD, and reduction in the scores of PI and PBI, due to regenerative and anti-inflammatory properties of i-PRF along with enhanced healing potential.

## Introduction

### Background

Periodontitis is a chronic, inflammatory disease caused by multiple factors that target the tooth supporting structures, including the gingiva, periodontal ligament, alveolar bone, and cementum. It is initiated by dysbiotic microbial biofilms and sustained by a destructive host immune response, ultimately resulting in pocket formation, clinical attachment loss (CAL), and resorption of alveolar bone. Irrespective of age, gender, and ethnicity, it affects individuals across all demographics and is driven by specific microorganisms or the group of microorganisms further responsible for the hard and soft tissue destruction of the periodontium. The new classification system has provided an enhancement in diagnosis and treatment by incorporating staging and grading criteria, which help practitioners in the assessment of disease severity and plan appropriate treatment. Stage II periodontitis, according to the current classification of periodontal and peri-implant diseases and conditions (2017), involves moderate symptoms, such as 5 mm probing depth (PD) and 3 to 4 mm CAL, involving horizontal bone loss without loss of tooth due to periodontitis. Stage III is more severe, with ≥6 mm PD, ≥5 mm CAL, ≥3 mm vertical bone loss, class II or III furcation involvement, and tooth loss due to periodontitis of less than or equal to 4 teeth [1]. Periodontal therapy primarily aims to halt the inflammation by minimizing the microbial load of periodontal pathogens. Its success largely depends upon the effective and complete removal of both subgingival and supragingival bacterial biofilms, smear layer, bacterial endotoxins, and contaminated radicular cementum [2,3].

Periodontal disease could be managed by performing quadrant-wise scaling and root planing (SRP) in multiple 7‐ to 14-day spaced visits [4]. This conventional strategy of management of periodontal disease was reevaluated when the concept of full-mouth disinfection (FMD) was introduced in the early 1990s [5]. FMD is a treatment approach for periodontitis that aims to manage periodontal pockets and prevent reinfection from other reservoirs of bacteria in the oral cavity, such as the tongue, tonsils, and mucous membrane. It involves performing SRP in a single session across the entire mouth, using antibacterial agents such as chlorhexidine. The effectiveness of this method has been evaluated in multiple clinical studies [6]. Chlorhexidine adheres well to teeth and oral tissues, showing high substantivity and exhibiting minimal irritation. Several investigations resulted in a reduction of bacterial cross-contamination after performing full-mouth SRP along with disinfection within the span of 24 hours and have offered better clinical results and microbiological outcomes compared to traditional stepwise treatment protocol [7-9].

Platelet concentrates have gained much popularity in regenerative dentistry and medicine due to their excellent healing properties and ability to promote neovascularization. While platelet-rich plasma (PRP) is obtained after centrifugation of blood with the inclusion of anticoagulants which was found to impair cellular inflammatory activation, ultimately delaying the healing process and compromising the regenerative potential, the newer generation of platelet concentrates called platelet-rich fibrin has eliminated the use of anticoagulants showing superior wound healing when compared with PRP, as demonstrated in numerous studies [10]. Injectable platelet-rich fibrin (i-PRF) was discovered by using the low-speed centrifugation concept, in which the quality, applicability, and effectiveness of platelet-rich fibrin were enhanced by reducing centrifugation speed [11].

The platelet-rich fibrin in an injectable form is liquid in consistency, rich in fibrinogen and thrombin, that remains in an injectable form for 15 to 20 minutes before converting into a fibrin matrix. It offers more sustained and controlled release of growth factors than PRP, leading to enhanced healing outcomes. It can be used in periodontal pockets and in periodontal regenerative procedures by mixing with bone grafts to create “sticky bone” to improve the stability of the graft. There are many studies showing its better regenerative potential, enhanced cell migration, higher transforming growth factor-β and collagen expression, antimicrobial properties, reducing inflammation, and promoting regeneration of tissues in gingival and periodontal therapy [12].

One-stage full-mouth disinfection (OS-FMD) with chlorhexidine is an established protocol; its outcomes are limited to antimicrobial effects without significant regenerative potential. The addition of i-PRF to the therapy will introduce a biologically active, autologous adjunct capable of delivering growth factors and regenerative cells in a minimally invasive manner. Integration of i-PRF into the OS-FMD protocol for stage II and III periodontitis, our study seeks to address the gap between infection control and tissue regeneration.

### Primary Objectives

To evaluate and compare the outcomes of i-PRF as an adjuvant in OS-FMD and OS-FMD therapy alone by assessment of clinical parameters in patients with stage II and stage III periodontitis after 3 and 6 months in terms of reduction in PD and gain in CAL.

### Secondary Objectives

To evaluate and compare the outcomes of i-PRF as an adjuvant in OS-FMD and OS-FMD therapy alone by assessment of changes in the scores of plaque index (PI) and papillary bleeding index (PBI) post therapy in patients with stage II and stage III periodontitis after 3 and 6 months.

## Methods

### Trial Design

This study is designed as a randomized controlled prospective parallel-arm clinical trial to enable a direct comparison between the clinical outcomes of i-PRF as an adjuvant in OS-FMD therapy and OS-FMD therapy alone in patients with stage II and III periodontitis.

### Setting of the Study

In this study, approximately 30 healthy patients will be selected with no history of systemic disease, with stage II and III periodontitis. Recruitment of the patients will be conducted from the Outpatient Department of Periodontics at Sharad Pawar Dental College, Sawangi (Meghe), Wardha. Prior to participation, information about the study design, purpose, and methodology will be provided to all subjects and written informed consent will be obtained. Eligibility of the participants will be based upon specific inclusion criteria.

The elaboration of the protocol is according to the SPIRIT (Standard Protocol Items: Recommendations for Interventional Studies) guidelines and using the SPIRIT checklist and was registered at Clinical Trials Registry India (CTRI/2025/06/088718).

### Eligibility Criteria and Recruitment

Following clinical evaluation, 30 systemically healthy patients in total, with stage II and III periodontitis, must meet the criteria precisely listed in Textbox 1.

Textbox 1. **Inclusion and exclusion criteria for eligibility of the patients in the study.**

**Inclusion criteria**

- Generalized interproximal attachment loss affecting at least 3 teeth, excluding incisors and first molars
- Periodontal tissue destruction disproportionate to the amount of microbial deposits present
- Patients presenting with at least 20 natural teeth during examination
- Patients exhibiting probing depth and clinical attachment loss ≥4 mm at 2 or more sites of 12 teeth minimum
- The participants in the study should be healthy and free from any systemic condition
- Patients who had not undergone periodontal therapy within the past 6 months and were not undergoing or had not recently received antibiotic therapy
- Patients showing motivation and willingness to undergo therapy and comply with follow-up, respectively

**Exclusion criteria**

- Patients with known or suspected hypersensitivity or allergy
- Patients who had received the periodontal therapy in the past 6 months
- Patients presenting with any systemic disorder known to influence periodontal health
- Patients presenting with active infectious conditions other than periodontitis
- Patients with a habit of chronic alcoholism
- Patients having a history of tobacco consumption in any form, including smoking
- Immunocompromised individuals and those presenting with systemic health conditions
- Pregnant or lactating women

### Outcomes

One week after performing OS-FMD therapy, subgingival delivery of i-PRF will be done in all periodontal pockets in the test group. Both groups will be followed up after 3 months and 6 months for clinical evaluation to assess the outcomes of the therapy. Reduction in PD and gain in CAL will be recorded using the University of North Carolina 15 mm (UNC-15) periodontal probe, PI, according to the Turesky–Gilmore–Glickman modification of the Quigley Hein Index (1970) and PBI according to the index given by Rebelo and Queiroz [13].

### Sample Size Calculation

The formula used for sample size calculation will be n ≥[(Z_(1-α/2)+Z_(1-β))² * (σ₁²+σ₂²/r)] / (μ₁-μ₂)², with given values: Z_(1-α/2) for a 2-tailed 95% confidence level=1.96 and Z_(1-β) for 99% power=2.33, where mean (μ1) and SD (σ1) in group 1 will be 4.36 and 0.87 respectively, mean (μ2) and SD (σ2) in group 2 will be 3.04 and 0.66 respectively, and ratio (r)=1. The required sample size is 13 participants per group (n=13).

The sample size was determined on the basis of earlier studies involving the evaluation of clinical parameters in OS-FMD therapy. Considering the outcome variable of PD and the mean difference reported in existing literature, a sample size of 26 participants in total (13 participants per group) was considered to be adequate to achieve sufficient statistical power [6].

**Figure 1.**
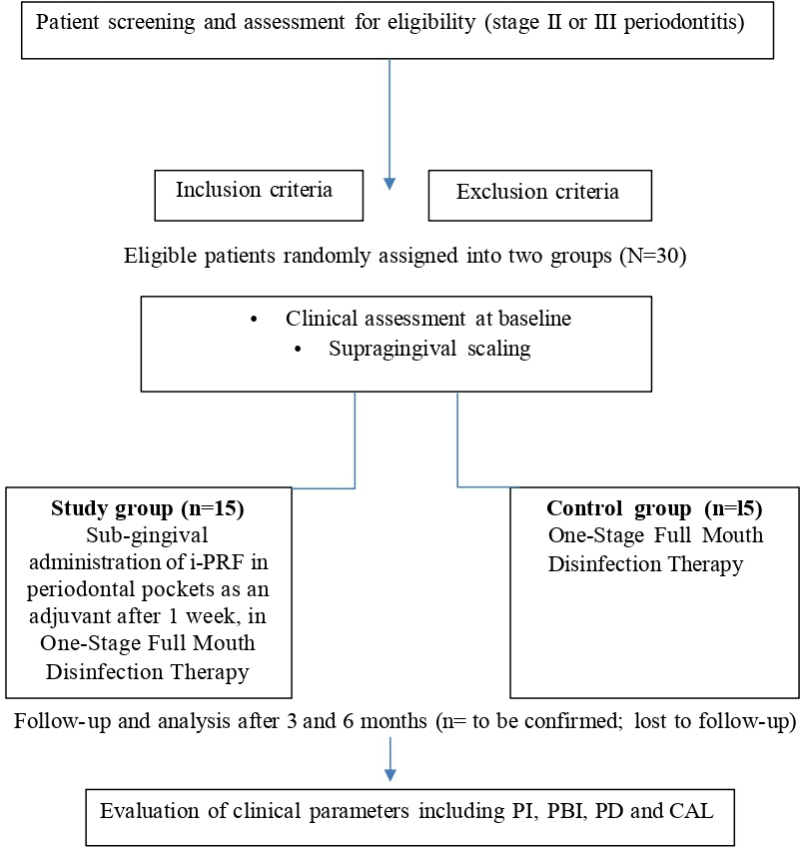
A simplified outline of the study protocol based on the CONSORT (Consolidated Standards of Reporting Trials) diagram. CAL: clinical attachment loss; i-PRF: injectable platelet-rich fibrin; PBI: papillary bleeding index; PD: probing depth; PI: plaque index.

To uphold the scientific validity of the study, the sample size was increased to 15 participants per group. This decision was made in anticipation of potential dropouts due to unforeseen reasons or noncompliance from the side of patients with follow-up protocols. By adopting this adjusted sample size, the study aims to preserve adequate statistical power, ensuring the reliability and integrity of the final outcomes, even in the event of participant loss (Figure 1).

### Recruitment

Patients in the Outpatient Department of Periodontics and Implantology, Sharad Pawar Dental College and Hospital, Sawangi (Meghe), Wardha, were recruited.

### Randomization and Masking

The eligible patients will be randomly assigned to either test group (i-PRF as an adjuvant in OS-FMD) or the control group (OS-FMD alone) in patients with stage II and III periodontitis. All participants will undergo a periodontal health assessment via clinical examination. Informed consent will be obtained from all participants in written format following a thorough explanation of the nature, purpose, potential benefits, and risks of the intervention. Along with obtaining informed consent, a complete and detailed medical history will also be obtained from the patients after receiving a thorough explanation of the proposed intervention.

Central randomization will be carried out for this study by using a secure computerized system, with assigning a unique study code to each participant, in order to ensure confidentiality. Data presented to the operators and research evaluators will be anonymized and presented using this coded format. During the phase of data analysis, the statistician will remain blinded to group allocation to minimize bias and enhance the objectivity and reliability of the results.

### Clinical Procedure

Information concerning dietary habits, systemic health status, oral hygiene practices, and gingival and periodontal conditions, and other routine clinical parameters, will be documented in the specially designed clinical chart, along with obtaining thorough medical and past dental history from the patients. Clinical examination of the patients will be performed under adequate illumination using a mouth mirror and a UNC-15 periodontal probe.

### Test Group

In the test group, patients will receive OS-FMD 1 week after the completion of supragingival scaling to allow for initial plaque control and gingival stabilization. Under local anesthesia, full-mouth subgingival SRP will be performed using an ultrasonic scaler and curettes, respectively, within 24 hours. Mechanical debridement will be complemented by the application of chlorhexidine to disinfect the intraoral niches.

The patient will be instructed to brush the dorsum of the tongue with 1% chlorhexidine gel for 1 minute, immediately following each instrumentation session. Tongue cleaning will be followed by rinsing with 0.2% chlorhexidine solution 2 times, each lasting for a minute. The pharyngeal region will then be sprayed with 0.2% chlorhexidine spray, followed by irrigation of all the periodontal pockets with 0.2% chlorhexidine solution thrice, within 10 minutes. Additionally, the prescription to use 0.2% chlorhexidine mouthwash twice daily will be given to the patient for 1 week following FMD [14]. One week postoperatively, i-PRF will be administered subgingivally into all periodontal pockets.

### Preparation of I-PRF

The preparation of i-PRF will be conducted in a sterile environment with careful handling to maintain biosafety. Blood will be withdrawn only once per patient in the test group**,** with no repeated phlebotomy, thereby minimizing participant discomfort and procedural risks. All phlebotomy procedures will be performed by trained personnel under strict aseptic conditions using sterile, disposable equipment to minimize infection risk. Participants will be closely monitored during and after venipuncture, and standard protocols for managing potential complications such as hematoma, vasovagal episodes, or allergic reactions will be in place. In case of emergency, immediate medical management will be carried out and referral, if necessary, and the case will be reported to the Data Monitoring Committee (DMC) and the Institutional Ethics Committee (IEC) according to the severity of adverse events (AEs).

10 ml of venous blood will be drawn immediately, just before subgingival administration, through antecubital venipuncture under strict aseptic conditions. i-PRF is a second-generation platelet concentrate, which is a blood-derived autologous biomaterial that possesses a 3D fibrin network, while maintaining its fluid consistency [15]. The obtained blood without the use of anticoagulants is then transferred into a sterile glass test tube. The whole blood will be subjected to low-speed centrifugation at 700 rpm (60×g) at room temperature for 3 minutes using the Dental Laboratory Centrifuge by Labtech Disposables. The low-speed centrifugation technique allows the separation of the components of blood, allowing the formation of the upper layer of liquid, which is rich in fibrinogen and growth factors, collected as i-PRF. In contrast to PRP, which requires multiple centrifugation steps and the addition of anticoagulants, for i-PRF, a single spin is required without the use of anticoagulants. This allows preservation of higher concentrations of growth factors and regenerative cells, enhancing its efficacy in the promotion of tissue regeneration and healing [12].

### Control Group

In the control group, patients will receive OS-FMD 1 week after the completion of supragingival scaling. Under local anesthesia, full-mouth subgingival SRP will be performed using an ultrasonic scaler and curettes, respectively, within 24 hours. Mechanical debridement will be complemented by the application of chlorhexidine to disinfect the intra-oral niches.

The patient will be instructed to brush the dorsum of the tongue with 1% chlorhexidine gel for 1 minute, immediately following each instrumentation session. Tongue cleaning will be followed by rinsing with 0.2% chlorhexidine solution 2 times, each lasting for a minute. The pharyngeal region will then be sprayed with 0.2% chlorhexidine spray, followed by irrigation of all the periodontal pockets with 0.2% chlorhexidine solution thrice, within 10 minutes. Additionally, the prescription to use 0.2% chlorhexidine mouthwash twice daily will be given to the patient for 1 week following FMD [14].

### Clinical Measurements

#### Clinical Indices

In order to ensure consistency, reduce interoperator variability, and minimize bias, all clinical recordings will be performed by the same calibrated examiner who is blinded to group allocation. The mean values of these indices will be calculated for outcome assessment.

The status of oral hygiene will be evaluated using the Full Mouth Plaque Index, based on the Turesky-Gilmore-Glickman modification of the Quigley Hein Index (1970) by which assessment of plaque will be carried out on the buccal or labial and lingual surfaces of all teeth after application of a disclosing agent [16].

For the measurement of gingival inflammation, the full-mouth PBI by Rebelo and Queiroz [13] will be recorded. Recordings will be obtained by carefully inserting the periodontal probe into the gingival sulcus at the base of the papilla on the mesial aspect, then gently moving it coronally toward the tip of the papilla. The procedure will be repeated on the distal aspect of the same papilla. The intensity of bleeding provoked by probing will be assessed using a standardized scale ranging from 0 to 4 to evaluate gingival inflammation [16].

#### Probing Measurements

To evaluate treatment outcomes, clinical parameters will be assessed using the UNC-15 probe (Hu-Friedy) to measure PD and CAL. For recording probing measurements, the deepest probing site will be evaluated and recorded. Measurements will be taken at baseline, and subsequently at 3 and 6 months.

### Statistical Analysis

The values of mean (SD) will be calculated for each of the clinical periodontal parameters, including PI, PBI, PD, and CAL. To assess the significance of observed differences using standard methods, statistical analysis will be performed. To compare baseline values of the findings to those values at 6 months within both groups, the Wilcoxon signed-rank test will be used. Comparisons of PI, PBI, CAL, and PD will be made at baseline, after 3 months of therapy, and then after 6 months. A *P* value less than .05 will be considered statistically significant, while a *P* value greater than .05 will be considered statistically nonsignificant.

### Ethical Considerations

Ethical approval for the study is obtained from the IEC (reference number DMIHER(DU)/IEC/2025/751) of Datta Meghe Institute of Higher Education and Research (deemed to be a university). The trial has been registered prospectively with the Clinical Trials Registry of India (CTRI registration no CTRI/2025/06/088718). All the participants will be provided with the sheet consisting of information regarding the purpose, process involved, and specifications of the study. The queries of participants will be addressed, if any. Informed consent in written format will be obtained from the participants before starting this trial. Written informed consent will be obtained from the patients for the publication of their data. The participants are free to withdraw at any time during the study. Clinical assessment will be done by taking aseptic precautions into consideration, without any harm to the patient. Collection of blood sample, transferring it into a glass test tube, and preparation of i-PRF will be done with aseptic precautions and expertise without any harm to the patient. The privacy and confidentiality will be protected by deidentifying the study data. Participants will not receive any compensation for their participation in this study.

## Results

The study received approval from the IEC in June 2025. Enrollment of participants began in September 2025 and will last up to March 2026. The study is scheduled to conclude by the end of 2026 after assessments and analyses. The accessibility of the study results is anticipated in early 2027. The results of this study will highlight the therapeutic efficacy of i-PRF as an adjunct to OS-FMD therapy, evaluated on the basis of clinical parameters including PI, PBI, PD, and CAL.

The dissemination plans for the study results are via multiple channels, including research reports, peer-reviewed publications, and presentations at national and international conferences. A primary paper, focusing on the outcomes aligned with the study objectives, will be submitted to a peer-reviewed journal with a high impact factor for publication.

## Discussion

### Anticipated Findings

Periodontal therapy plays a vital role in the prevention of progression of disease and inflammation of the gingiva and supporting periodontal structures, including the periodontal ligament and alveolar bone. If the condition is left untreated, it can cause progressive attachment loss, resorption of alveolar bone, tooth mobility, and ultimately loss of the tooth. The primary aim of periodontal therapy is to stop the disease from progressing by eliminating microbial load in the form of biofilm or calculus, controlling inflammation, and restoring periodontal health through nonsurgical therapy initially and surgical interventions further, if required. OS-FMD provides a comprehensive approach by treating the entire oral cavity within 24 hours, targeting not only periodontal pockets but also other oral niches in order to prevent cross-contamination and reinfection. The OS-FMD results in a reduction of the overall treatment time and microbial load more efficiently than conventional quadrant-wise therapy. The therapy leads to enhancement of patient compliance, limits the recolonization of pathogens between treated and untreated sites, and serves as an economical option. Over 2 decades of research have shown that FMD can achieve clinical outcomes comparable to or better than conventional approaches, especially when tailored with adjunctive therapies. Chlorhexidine was most effective as an adjuvant, especially when combined with FMD [5].

Bollen et al [17] in their pilot study compared standard periodontal treatment comprising multiple sessions of SRP with FMD in patients with chronic periodontitis. Intensive scaling, subgingival irrigation, and tongue brushing using 1% chlorhexidine gel and 0.2% chlorhexidine rinses within 24 hours were carried out in the test group and simply SRP in the control group, followed by oral hygiene instructions to both groups. Plaque samples showed a greater reduction of harmful bacteria in the test group, especially around multi-rooted teeth, lasting up to 8 months. Anaerobic microbial flora remained lower in the test group than in the control. A significant increase in beneficial microflora was observed in the test group up to 4 months. FMD showed superior short-term microbiological outcomes.

Stein et al [18] in their randomized clinical trial compared four treatment groups: quadrant scaling and root planing (Q-SRP), full-mouth scaling, FMD, and FMD with erythritol air-polishing (FMDAP). All methods resulted in a reduction in PD and gain in CAL, along with improvement in the values of Balance of Payments over the period of 6 months. The FMDAP group showed the greatest reduction in PD for both moderate and deep pockets compared to Q-SRP. FMD also performed better than Q-SRP in deep pockets, but not as well as FMDAP. Conclusively, all the full-mouth approaches were more time-efficient than Q-SRP, with FMDAP being most effective in terms of pocket closure, especially in single-rooted teeth.

Platelet concentrates have gained popularity in the field of periodontal regeneration due to their wide range of use. Along with having the advantage of being autologous, it resulted in enhanced soft and hard tissue healing and amplified regenerative potential with uncomplicated preparation techniques. Differences in the preparation protocol have led to variable results in the literature [19].

i-PRF is a second-generation platelet concentrate that has shown significant regenerative potential in the field of dentistry and medicine. i-PRF differs from traditional PRP in that it is prepared without the use of anticoagulants, enabling a sustained and gradual release of growth factors, such as platelet-derived growth factor, transforming growth factor-β, and vascular endothelial growth factor. This promotes enhanced cell proliferation, migration, angiogenesis, and tissue regeneration, leading to overall enhancement in clinical parameters [10].

Thamaraiselvan et al [20] in their randomized parallel arm clinical trial evaluated the clinical parameters, such as PI, PBI, PD, and CAL, along with microbiological assessment at baseline, 6 weeks, and 12 weeks in patients with periodontitis randomly allocated into 3 groups with group A receiving ciprofloxacin-loaded i-PRF after SRP, group B receiving local drug delivery with i-PRF, and group C receiving SRP alone. The results demonstrated greater improvements in clinical parameters and a reduction in the microbial load as compared to other groups. The study concluded that ciprofloxacin-loaded i-PRF is a promising, autologous, and biocompatible vehicle for local drug delivery, combining antimicrobial efficacy of ciprofloxacin with regenerative potential, favorable tissue integration capability of i-PRF and its liquid injectable form offering the additional advantage of being flowable at placement before slowly polymerizing to gel in situ, thus adapting well to periodontal pocket. anatomy and minimizing the chances of displacement, thereby enhancing periodontal healing outcomes beyond SRP alone.

Miron et al [12] stated the natural clotting tendency of i-PRF post injection and effective delivery in periodontal pockets due to its injectable form, where it can demonstrate superior outcomes in terms of reduction in pocket depth, periodontal regeneration, gain in CAL, wound healing, and gingival tissue thickening. It also exhibits antimicrobial and anti-inflammatory properties, modulating polarization of macrophages toward a pro-healing M2 phenotype.

In this study, we hypothesize that the incorporation of i-PRF as an adjunct to OS-FMD will result in superior improvements in clinical outcomes such as clinical attachment gain and PD reduction, along with decreased PI and PBI scores due to its regenerative and anti-inflammatory properties along with enhanced healing potential, beyond the antimicrobial benefits achieved with chlorhexidine in the standard OS-FMD protocol. In addition, the preparation of i-PRF is a cost-effective, clinically feasible, minimally invasive, autologous, chairside procedure that can be realistically integrated into routine clinical practice.

The outcomes of this study will throw light on the therapeutic efficacy of an adjunctive use of i-PRF to OS-FMD therapy. Current protocols available in the literature for OS-FMD in the management of periodontitis primarily recommend SRP with the use of antimicrobials such as chlorhexidine according to the standard protocol given by Quirynen and colleagues [21]. By incorporating i-PRF, our trial will explore a regenerative adjunct that could potentially enhance healing outcomes and reduce disease recurrence. Integrating such biologically based approaches into established frameworks may provide clinicians with a more comprehensive, evidence-supported strategy, bridging antimicrobial therapy with regenerative potential. We believe that by establishing strong clinical evidence first, our study will provide a sound basis for future research to explore microbiological and biochemical pathways in greater detail. We also highlight that while this trial focuses on systemically healthy patients, the protocol may serve as a foundation for future studies in more diverse populations, thereby broadening its clinical relevance. The overall findings of the study will provide a strong groundwork for broader and more mechanistic investigations in the future.

### Declarations

#### Oversight and Monitoring

Composition of the coordinating center and trial steering committee consisting of the following: (1) the head of the department of periodontics, (2) postgraduate guide, (3) research scientist, (4) principal investigator, (5) statistician, and (6) data manager.

#### Composition of the DMC, its Role, and Reporting Structure

The composition of DMC is as follows: senior faculty members who are not directly involved in this trial and 1 external expert in periodontology.

#### AE Reporting and Harms

All AEs will be actively monitored and documented throughout the study. Mild, self-limiting events such as transient discomfort or swelling will be managed conservatively and recorded in the case report forms. Any moderate or severe AEs will be immediately reported to the principal investigator and the DMC, with appropriate medical care provided. Serious AEs, if any, will be reported to the IEC within 24 hours, and participants will be withdrawn from the study if deemed necessary for safety.

#### Frequency and Plans for Auditing Trial Conduct

The trial conducted will be reviewed by the project management group that meets every month. The trial steering group and the independent data monitoring and ethics committee will meet to review and conduct the trial period until the trial is complete.

#### Dissemination Plans

Dissemination plans include publication, research reports, presentations at national and international conferences, and results will also be shared with professional organizations and guideline committees to support translation into future periodontal practice recommendations.

### Conclusions

This study highlights the clinical effectiveness of i-PRF as an adjunct to OS-FMD in the management of periodontitis. By comparing adjunctive use of i-PRF in OS-FMD with OS-FMD therapy alone, the study aims to assess improvements in clinical parameters, including CAL, PD, PI, and PBI. The findings of the study are expected to provide evidence regarding the potential regenerative and anti-inflammatory benefits of i-PRF, thereby contributing to improved therapeutic strategies and clinical decision-making in periodontal care.

## Supplementary material

10.2196/81032 Checklist 1 SPIRIT Checklist.

## Abbreviations

AE: adverse event
CAL: clinical attachment loss
DMC: Data Monitoring Committee
FMD: full mouth disinfection
FMDAP: full-mouth disinfection with erythritol air-polishing
i-PRF: injectable platelet-rich fibrin
IEC: Institutional Ethics Committee
OS-FMD: one-stage full-mouth disinfection
PBI: papillary bleeding index
PD: probing depth
PI: plaque index
PRP: platelet-rich plasma
SPIRIT: Standard Protocol Items: Recommendations for Interventional Studies
SRP: scaling and root planing
UNC-15: University of North Carolina 15 mm periodontal probe
